# A chromosome-scale genome assembly of the nipa palm hispid beetle *Octodonta nipae*

**DOI:** 10.1038/s41597-024-03417-7

**Published:** 2024-05-30

**Authors:** Baozhen Tang, Chuanlin Yin, Kang He, Shaomin Tao, Lang Fu, Ying Liu, Fei Li, Youming Hou

**Affiliations:** 1 State Key Laboratory of Ecological Pest Control for Fujian and Taiwan Crops, Fujian Agriculture and Forestry University, Fuzhou, 350002 China; 2 Key Lab of Biopesticide and Chemical Biology, Ministry of Education & Fujian Provincial Key Laboratory of Insect Ecology, Department of Plant Protection, Fujian Agriculture and Forestry University, Fuzhou, 350002 China; 3 Zhejiang Provincial Key Laboratory of Biometrology & Inspection & Quarantine, College of Life Sciences, China Jiliang University, Hangzhou, 310018 China; 4 State Key Laboratory of Rice Biology & Ministry of Agricultural and Rural Affairs Key Laboratory of Molecular Biology of Crop Pathogens and Insects & Key Laboratory of Biology of Crop Pathogens and Insects of Zhejiang Province, Institute of Insect Sciences, Zhejiang University, Hangzhou, 310058 China; 5 Key Laboratory of Green Prevention and Control of Agricultural Transboundary Pests of Yunnan Province and Agricultural Environment/ Agriculture Environment and Resources Institute, Yunnan Academy of Agricultural Sciences, Kunming, 650205 China; 6 Present Address: Hunan Horticulture Research Institute, Hunan Academy of Agricultural Sciences, Changsha, 410125, Hunan Province, China

**Keywords:** Entomology, Genome evolution

## Abstract

Nipa palm hispid beetle (*Octodonta nipae*) is an insect species that is native to Malaysia but has spread to southern China and beyond, seriously threatening palm production. A lack of high-quality genome resources has hindered understanding of the insect’s invasive characteristics and ecological adaptations. Here, we combined Illumina short read, PacBio long-read, and high-throughput chromosome conformation capture (Hi-C) sequencing technologies to generate a high-quality, chromosome-scale genome assembly of nipa palm hispid beetle. The genome assembly was 1.31 Gb in size, consisting of nine chromosomes. The contig and scaffold N50 values were 1.022 Mb and 148.6 Mb, respectively. The genome assembly completeness was estimated at 99.1%. Annotation revealed 16,305 protein-coding genes and 62.16% repeat sequences. This high-quality genome assembly is a valuable resource that will contribute to understanding of the genetic factors underlying the invasive characteristics of nipa palm hispid beetle, ultimately promoting development of efficient control policies.

## Background & Summary

Nipa palm hispid beetle (*Octodonta nipae*) belongs to the Cryptonychini tribe of the superfamily Chrysomeloidea and is a serious pest of palm plants. These beetles can have devastating impacts on humans because palm plants are important sources of food and economic value in tropical and semitropical regions^1–3^. *O. nipae* is native to Malaysia, but was detected in Hainan Province, China, in 2001^4^. Since then, it has rapidly spread to other provinces, including Guangdong, Guangxi, Fujian, and Yunnan, which are the primary palm-producing regions in China^5^. *O. nipae* is now found in most southern provinces of China and has spread further abroad from Malaysia, having been detected even on the island of Cyprus^6^.

*O. nipae* is a gregarious species; larvae and adults typically assemble in the same region of a palm plant. They feed and dwell only in fronds attached to the central shoot. Most adults are found in the young fronds, whereas larvae are detected only in very tightly furled fronds and trunk fibers. Infested palms display necrotic patches, appearing as longitudinal white streaks, on young leaflets. Sustained attack results in brown patches and streaks of various sizes as the fronds open. Young leaves become shrivelled and curled, then the plant dies. *O. nipae* can infest many palm species, including queen palm (*Syagrus romanzoffiana*), Canary island date palm (*Phoenix canariensis*)^5,7^, Chinese windmill palm (*Trachycarpus fortune*)^8–10^, areca palm (*Areca catechu*), and coconut palm (*Cocos nucifera*)^11,12^. Due to this wide host range, *O. nipae* is a threat to the palm planting industry, the ornamental industry, and the ecological environment^5^.

At present, chemical control remains the major strategy for *O. nipae* management^5^. However, because the beetle lives cryptically and palm plants usually have high stems (i.e., above human reach), the chemical control efficiency is relatively low. In other pest species, genomic resources have proven beneficial in developing novel control strategies. The lack of a high-quality *O. nipae* genome assembly has hindered deep understanding of this notorious insect pest. To address this issue, we here generated a high-quality chromosome-scale *O. nipae* genome assembly using a combination of Illumina short reads, PacBio high fidelity (HiFi) reads, and high-throughput chromosome conformation capture (Hi-C) data. This resource is expected to contribute to future development of control measures for an economically devastating invasive insect species.

## Methods

### Sample collection

*O. nipae* individuals were selected from a laboratory population maintained at Fujian Provincial Key Laboratory of Insect Ecology. The progenitors of the population were collected from *P. canariensis* in Zhangzhou, Fujian Province, in 2017. Insects were fed fresh *P. canariensis* leaves and maintained in a growth chamber at 25 ± 1 °C with 80% ± 5% humidity under a 12/12 h light/dark photoperiod.

### Flow cytometry

Samples were prepared by collecting the heads of adult female *O. nipae* into 1.5 ml centrifuge tubes, with 14 heads per tube. These were mechanically disrupted using a pestle in 200 μl of Galbraith’s nuclear dissociation solution. The homogenized tissue was then filtered through a 38 μm nylon mesh to extract the nuclear suspension, which was centrifuged at 1000 r/min for 5 minutes at 4 °C. The resulting nuclear pellet was resuspended in 400 μl of 1 × PBS and gently agitated to ensure thorough mixing. For staining, the nuclei were treated with PI solution (final concentration: 50 μg/ml) and RNase A (final concentration: 20 μg/ml), and incubated at 4 °C in the dark for 5–20 minutes. Flow cytometry analysis revealed that the DNA content of the *O. nipae* nuclei was 7.5 times higher than that in the reference species, *Drosophila melanogaster* Canton-S strain adults (genome size: 176.4 Mb). This comparison indicates an estimated genome size of approximately 1.32 Gb for *O. nipae* (Figure S1).

### Illumina sequencing and genome survey

Genomic DNA (gDNA) was extracted from nearly 15 adult female *O. nipae* using the QIAamp DNA Mini Kit (QIAGEN, Valencia, CA, USA). The purity and quantity of gDNA were determined using NanoPhotometer (IMPLEN, CA, USA) and Qubit^®^ 3.0 Flurometer (Life Technologies, CA, USA) instruments, respectively. After quality control, gDNA was sheared to ~350-bp fragments using an Annoroad^®^ Universal DNA Fragmentase kit V2.0, and a paired-end gDNA library was prepared with the Annoroad^®^ Universal DNA Library Prep Kit (V2.0) following the manufacturer’s protocol. The library was sequenced by Annoroad Gene Technology (Beijing), Co., Ltd. on a HiSeq X-Ten platform (Illumina, San Diego, CA, USA) to generate paired-end 150-bp reads. The raw reads were quality-filtered with fastp (v0.23.2)^13^ to remove the following reads: (1) those with adapter contamination (containing >5 bp of adapter sequence); (2) those with low sequencing quality (≥15% of bases with Q-values < 19); (3) those enriched in unknown bases (N bases >5%); and (4) those for which the paired read was eliminated in steps 1–3. Quality filtering yielded a total of 85.25 Gb of clean Illumina reads (Table 1). A genome survey and K-mer analysis were employed to estimate the genome size, repeat content, and heterozygosity using Jellyfish (v1.0.0)^14^ and GCE (v1.0.2)^15^. The genome size of *O. nipae* was estimated to be 1.23 Gb with 45.71% repeat sequence content and 0.62% heterozygosity at K = 21 (Table S1 and Figure S2).

**Table 1:** Statistics of the sequencing data.

Library type	Platform	Data size (Gb)	Depth (X)	Average length (bp)
WGS short reads	Illumina HiSeq X-Ten	85.25	~65	150
WGS long reads	Pacbio Sequel II	160.37	~120	35,707
Hi-C	Illumina HiSeq X-Ten	98.76	~75	150
RNA-Seq	Illumina HiSeq X-Ten	6	—	100
Male WGS	Illumina HiSeq X-Ten	45.64	~35	150
Female WGS	Illumina HiSeq X-Ten	41.38	~32	150

**Table 2:** Comparison of genome assemblies in five Coleoptera species.

Features	*Octodonta nipae*	*Dendroctonus valens*	*Dendroctonus ponderosae*	*Tribolium castaneum*	*Aethina tumida*
Genome size (Mb)	1,310.80	322.41	252.85	165.94	259.9
Karyotype	8 + XO	12 + XY	11 + XY	9 + XY	6 + XY
Number of contigs	2581	1144	59,583	7059	38
Number of scaffolds	536	923	8188	2149	—
Number of assembled chromosomes	9	14	NA	10	8
Genome assembly quality					
Contig N50 (kb)	1,022.37	985.47	7.45	73.05	11,742
Scaffold N50 (Mb)	148.60	1.66	0.63	4.46	—
Linkage group N50 (Mb)	148.60	24.4	NA	15.3	36.78
BUSCO genes (%)	99.1	95.2	95.3	99.3	99.5
Genomic features					
Repeat (%)	62.17	45.22	21.12	28.9	23.41
G + C (%)	33.54	36.7	28.7	35.19	27.93
Gene annotation					
Number of genes	16,305	13,751	14,342	16,590	14,581

### PacBio sequencing and genome assembly

gDNA was extracted from nearly 15 adult female *O. nipae* using the QIAamp DNA Mini Kit (QIAGEN), and the resulting gDNA integrity was determined with the Agilent 4200 Bioanalyzer (Agilent Technologies, Palo Alto, CA, USA). gDNA samples (15 µg each) were sheared into fragments of ~15 kb in size using g-Tubes (Covaris, Woburn, MA, USA). Fragments were purified using 0.45 × AMPure PB beads (Beckman Coulter, Brea, CA, USA) and size-selected (15–18 kb) using the Sage ELF system (Sage Science, Beverly, MA, USA). A SMRT bell library was constructed using specific primers, Sequel II DNA polymerase, and SMRT bell templates with the Pacific Biosciences SMRTbell Express Template Prep Kit 2.0. Sequencing was performed by Annoroad Gene Technology with 8 M SMRT cells on the Sequel II System (PacBio, Menlo Park, CA, USA). Self-correction with Canu (v2.2)^16^ produced 160.37 Gb of clean reads (Table 1). Three long-read genome assembly programs were used to assemble draft genomes from the PacBio and Illumina reads: Canu, FALCON (v0.3.0.)^17^, and SmartDenovo (v1.0)^18^. The three assemblies were compared for contig N50 length, assembly size, and completeness. The latter parameter was evaluated with Benchmarking Universal Single-Copy Orthologs (BUSCO) (v5.4.5)^19^ and the insect database (insect_odb10). The SmartDenovo assembly performed the best, with the longest contig N50 length and the highest BUSCO score, and was thus selected for subsequent analyses. The clean Illumina short reads were used to polish the assembly twice using Pilon (v1.23)^20^. The resulting draft genome was 1.31 Gb in size, consisting of 2,581 contigs with an N50 length of 1.02 Mb. The Illumina short reads were mapped to the draft assembly to evaluate the quality; 97.79% of the short reads were uniquely mapped to the assembly and the genome coverage rate was 95.05%, indicating that the assembled genome was of high quality (Figure S3).

### Hi-C sequencing and genome anchoring

gDNA was extracted from the cephalothoracic tissue of ~100 adult female *O. nipae* and used to construct a Hi-C library following the standard protocol^21^. Briefly, nuclear DNA was fixed with formaldehyde and digested with DpnII (New England Biolabs, UK). Biotinylated nucleotides were added to the termini of the fragmented DNA, then fragments of ~500 bp in size were collected through enrichment and size selection. The library was sequenced by Annoroad Gene Technology on the Illumina HiSeq X-Ten platform to generate paired-end 150-bp reads. This yielded 98.76 Gb of raw Hi-C data. After reads were quality-filtered as described above for the Illumina short-read sequencing data, the clean reads were mapped to the draft genome with BWA (v0.6.2)^22^. Clean paired-end reads that were uniquely mapped to the draft genome were selected for analysis if they were near restriction sites based on the Hi-C data. Scaffolds were clustered using a combination of ALLHIC (v1.0)^13,23^, 3D-DNA (branch 201008)^24^, Juicer (v1.6)^25^, and Juicebox (v2.20)^26^. Scaffold arrangement was validated based on interaction strengths between pairs of reads; the scaffold order was then manually checked and corrected. Orientations were assigned to each cluster group.

**Fig. 1:**
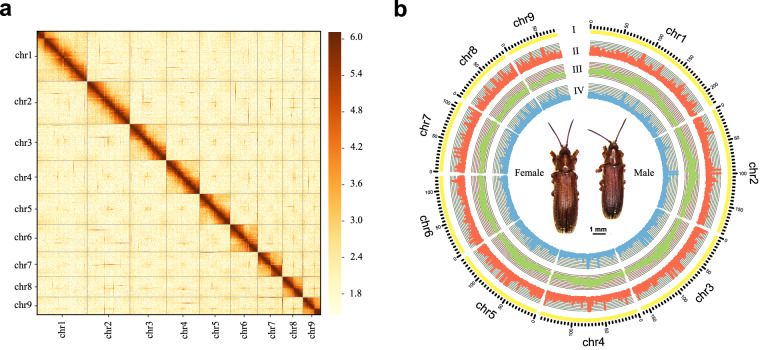
Heatmap of genomic Hi-C data and overview of the genomic landscape of nipa palm hispid beetle (*Octodonta nipae*). (**a**) Heatmap showing all interactions among the nine chromosomes of *O. nipae*. In general, intra-chromosomal interactions (blocks on the diagonal line) were strong, whereas inter-chromosomal interactions were weaker. Color indicates Hi-C interaction frequency. (**b**) Summary of nipa palm hispid beetle genome characteristics based on a sliding window size of 200 kb. The outermost to the innermost circle show the following: I. chromosome ideograms; II. protein-coding gene density; III. GC content density; and IV. repeat element density.

In total, 2,054 contigs (representing 96.15% of the draft genome assembly) were orientated and anchored to nine chromosomes, resulting in a scaffold N50 value of 148.60 Mb (Fig. 1a and Table 1). BUSCO analysis demonstrated that 99.1% of universally conserved genes could be successfully detected, of which 85.7% were single-copy genes and 13.2% were duplicates (Table S2). This suggested that the anchored genome was highly complete and of sufficient quality for further analyses.

### Analysis of sexual chromosome

Genomic DNA was extracted from individual single-headed adult males and females, followed by genome resequencing using the Illumina HiSeq X-Ten platform with a read length of 150 bp. The extraction, library construction, and sequencing methods mirrored those of previous Illumina genome sequencing endeavors. Subsequent to quality control, the resulting resequencing data amounted to 45.64 Gb and 41.38 Gb for male and female individuals, respectively. Clean reads underwent mapping to the genome utilizing BWA software, with genome coverage assessed using bedtools (v2.31.0)^27^ within 100 kb windows. Analysis of male-to-female coverage ratios plotted across each chromosome of the *O. nipae* genome revealed a distinctively female-biased coverage ratio (mean log2(male:female coverage) = −0.35) exclusively on chr1, indicative of an X sexual sequence. In contrast, other chromosomes exhibited nearly equal coverage between sexes (mean log2(male:female coverage) ≈ 0), consistent with autosomal expectations (Figure S4). Genome data for the red flour beetle (*Tribolium castaneum*), a coleopteran model species, were sourced from GenBank under accession number GCF_000002335.3. Genome-wide synteny between *O. nipae* and *T. castaneum* genomes was assessed using satsuma (v2.0) (https://github.com/bioinfologics/satsuma2), and collinearity plots were generated using circos (v0.69)^28^. Results demonstrated a pronounced collinearity between chr1 of *O. nipae* and the X chromosome of *T. castaneum* (Figure S5), supporting the inference that chr1 functions as the X sexual chromosome.

### RNA sequencing

Total RNA was extracted from 50 eggs, 30 larvae, 20 pupae, and 20 adults. Equal amounts of each sample type were combined to form a single RNA sample. RNA quality and quantity were assessed with a Bioanalyzer and spectrophotometer, respectively. An RNA sequencing library was prepared using the Illumina TruSeq RNA Library Preparation Kit. The quality and quantity of the resulting cDNA library was evaluated via quantitative PCR (qPCR) and a Bioanalyzer. The prepared library was sequenced by Annoroad Gene Technology on an Illumina HiSeq X-Ten platform to generate paired-end 150-bp reads.

### Genome annotation

Known tandem repeats were identified in the draft genome assemblies using Tandem Repeats Finder (TRF) (trf409)^29^. RepeatModeler (v2.0.4)^30^, which includes both RECON (v1.08)^31^ and RepeatScout (v1.0.6)^32^, was used to identify novel repeat sequences. Transposable elements (TEs) were predicted with rmblast (v2.14.0). Homology searches were conducted against RepBase (v26.03 library)^33^ using RepeatMasker (v4.1.5)^34^. Through this *ab initio* prediction and homology comparisons, ~62.17.8% of the nipa palm hispid beetle genome sequences were annotated as repeat elements (Fig. 1b). Short interspersed nuclear elements (SINEs), long interspersed nuclear elements (LINEs), long terminal repeats (LTRs), and DNA transposons accounted for 0.07%, 11.51%, 12.23%, and 11.97%, respectively, of the whole genome; 26.34% of repeat sequences were annotated as unclassified.

After masking repeat sequences, protein-coding and non-coding RNA (ncRNA) genes were classified using a combination of transcriptomic, homology searching, and *ab initio* prediction-based approaches. Homology-based annotation involved aligning reference protein sequences from 21 coleoptera species (Table S3), obtained from the National Center for Biotechnology Information (NCBI) database, against the *O. nipae* genome using TBLASTN (v2.2.29+) with an E-value threshold of 1E-5. Concatenation of all BLAST hits was performed using Solar software (v0.9.6), followed by extraction of genomic regions 1 Kb upstream and downstream of each candidate gene for precise gene structure prediction through GeneWise (v2.4.1)^35^. These predictions constituted the ‘Homology set.’ Transcriptome-based annotation utilized HISAT2 (v2.2.1)^36^ for read alignment and StringTie (v2.2.1)^37^ for transcript assembly, resulting in the ‘RNAseq-set’ of gene models. *Ab initio* gene prediction employed Augustus (v3.2.3)^38^, GlimmerHMM (v3.0.4)^39^, and SNAP (v2013-11-29)^40^, with parameters trained using intact open reading frame (ORF) gene models from the RNAseq-set via Transdecoder (v5.7.1). Integration of all gene models into a consensus set was achieved using EVidenceModeler (v2.1.0)^41^, with prioritization given to evidence types: RNAseq-set > Homology-set > Augustus > SNAP = GlimmerHMM. Furthermore, genes encoding proteins fewer than 50 amino acids in length, supported solely by ab initio evidence and exhibiting low expression levels (<1.0), were filtered out.

Three types of ncRNAs (transfer RNA [tRNA], ribosomal RNA [rRNA], and small nuclear RNA [snRNA]) were annotated. To identify these classes of ncRNAs, the draft genome was analyzed after removing protein-coding genes, other types of ncRNAs, and repeat sequences. tRNAscan-SE was employed with the parameters for eukaryotes to identify genes encoding tRNAs. rRNA genes from all invertebrate species were used as queries in BLASTN searches against the draft genome fragments at E-value < 1E-5 to classify rRNA genes. INFERNAL was used with the Rfam database (release 14.9) to identify snRNA genes. These analyses yielded a total of 16,305 protein-coding genes (Table 3), 114 small nucleolar RNA (snoRNA) genes, 872 tRNA genes, 497 rRNA genes, and 157 microRNA (miRNA) genes (Table 4).

**Table 3:** Classification of repetitive sequences in *O. nipae* genome.

Category	Number of elements	Ratio (%) in genome
SINEs	2,998	0.07%
LINEs	390,917	11.51%
LTR elements	202,019	12.23%
DNA elements	437,207	11.97%
Unclassified	1,029,646	26.34%

**Table 4:** Classification of non-coding RNAs in *O. nipae* genome.

Category		Number	Average length (bp)	Total length (bp)	% of genome
miRNA		157	114.96	18,049	0.001377
tRNA		872	75.44	65,786	0.005019
rRNA	18S	205	547.94	112,327	0.008569
	28S	244	617.07	150,565	0.011486
	5.8S	13	154.69	2,011	0.000153
	5S	35	117.71	4,120	0.000314
snRNA	CD-box	43	121.35	5,218	0.000398
	HACA-box	4	137.25	549	0.000042
	splicing	67	136.37	9,137	0.000697

## Data Records

The Illumina, PacBio, and Hi-C sequencing data used for the genome assembly, along with the transcriptome sequencing data used for genome annotation, have all been deposited in the NCBI Sequence Read Archive (SRA) under a single accession number, SRP460189^42^. The chromosomal assembly has been deposited to GenBank under accession number JAVLUE000000000^43^. The genome assembly and annotated genes have been deposited to the figshare repository^44^.

## Technical Validation

The genome assembly was 1.3108 Gb in size with a scaffold N50 value of 148.60 Mb. The true genome size was generally consistent with the estimated size based on K-mer analysis (Figure S2). and the flow cytometry experiment (Figure S1). The Hi-C heatmap revealed a well-organized interaction pattern along the diagonal and surrounding the chromosome inversion region (Fig. 1a), supporting the accuracy of the chromosome assembly. BUSCO assessment successfully identified 99.1% of universally conserved genes based on the insecta_odb10 database (Table 2, Table S2), suggesting a remarkably complete *O. nipae* genome assembly.

### Supplementary information

Supplementary materials

## Data Availability

Programs used in data processing were executed with the default parameters except where otherwise specified in the Methods. No custom code was used in these analyses.
