# Supplementary material for: A chromosome-scale genome assembly of the nipa palm hispid beetle Octodonta nipae
Source: Sci Data. 2024 May 30;11:562. doi: 10.1038/s41597-024-03417-7 (PMC11139935; doi:10.1038/s41597-024-03417-7)
Supplement: Supplementary file 1 — Supplementary materials [file 41597_2024_3417_MOESM1_ESM.docx]

**Contents**

| **Title** | **Page Number** |
| --- | --- |
| Figure S1 | 2 |
| Figure S2 | 3 |
| Figure S3 | 4 |
| Figure S4 | 5 |
| Figure S5 | 6 |
| Table S1 | 7 |
| Table S2 | 8 |
| Table S3 | 9 |


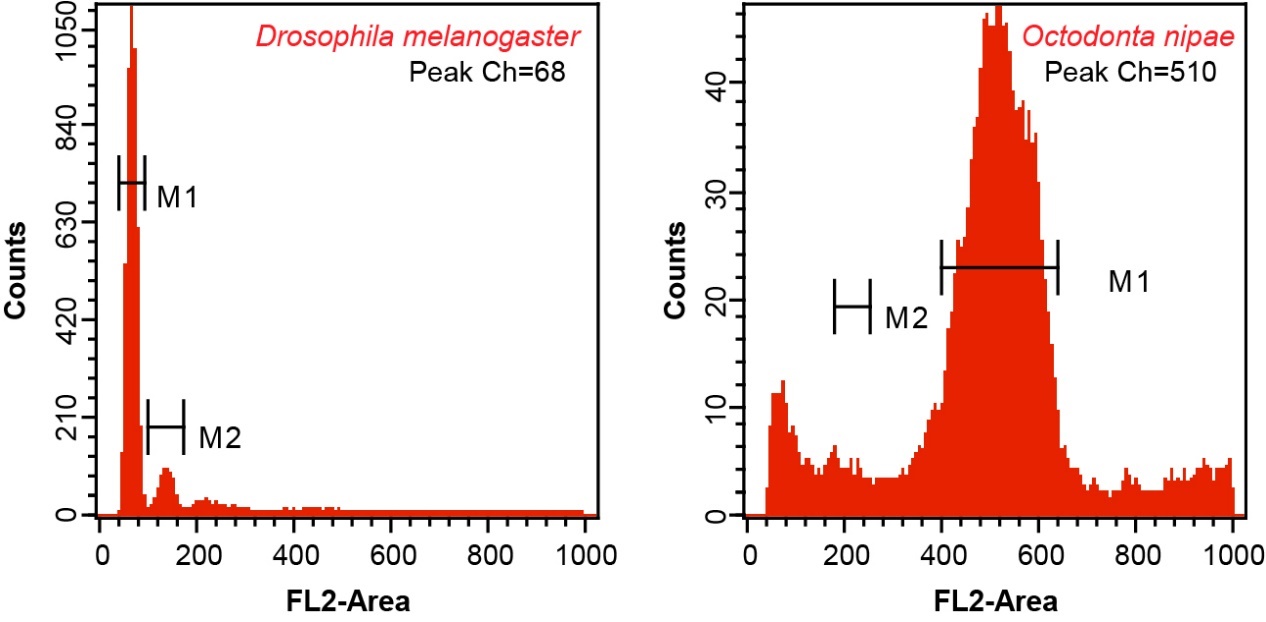


**Figure S1 Estimation of genome size in *O. nipae* using flow cytometry.**

# Figure S2 Distribution of K-mer frequencies in Illumina paired-end reads from K-mer anlysis.


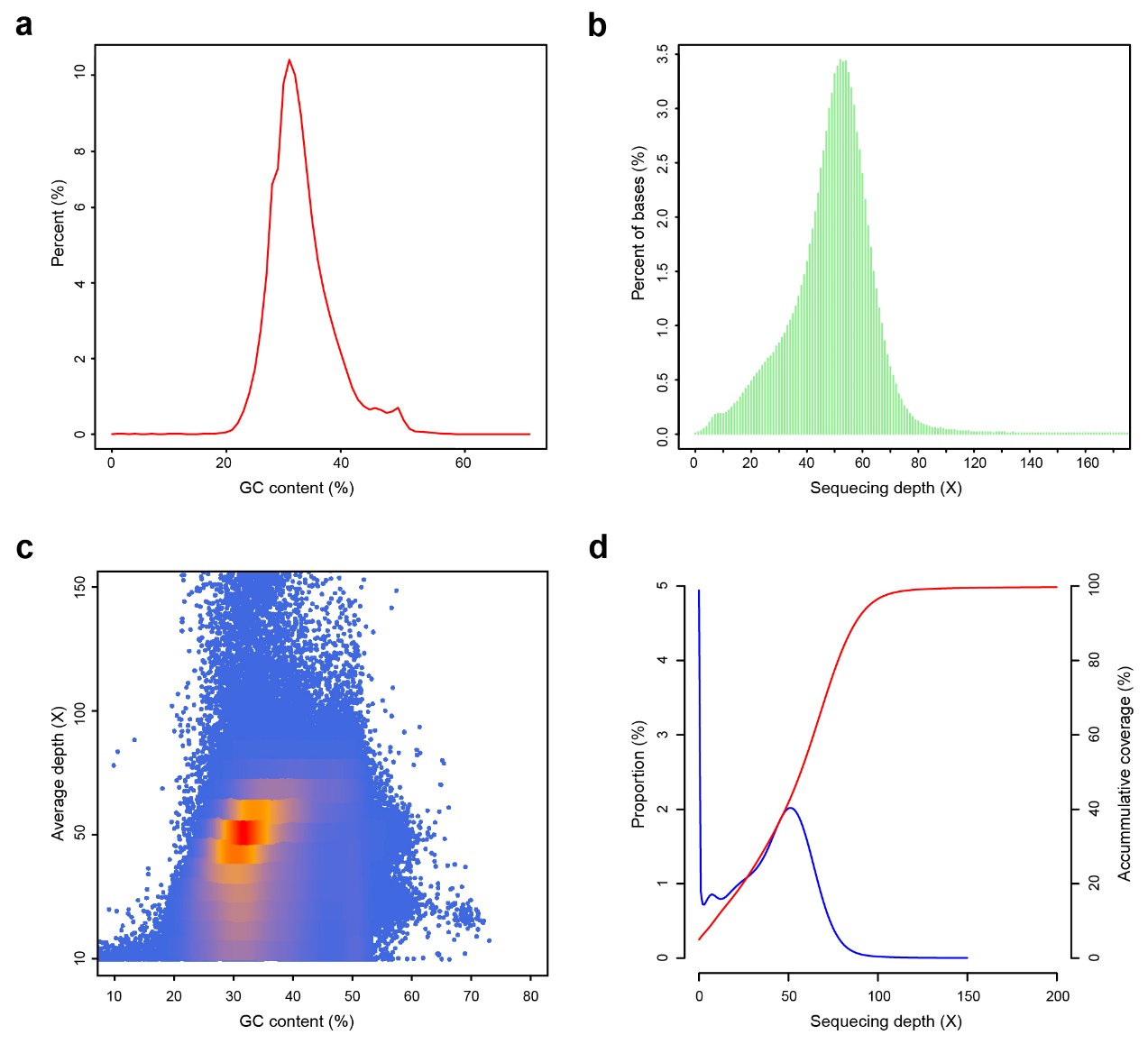


# Figure S3 Assessment the genome assembly of *O. nipae.*

(a) GC content distribution of the assembled genome in non-overlapping 10 kb windows. (b) Sequencing depth of the assembled genome in non-overlapping 10 kb windows. (c) Scatter distribution plot between GC content and sequencing depth in non-overlapping 10 kb windows. (d) Schematic diagram illustrating genome read coverage depth distribution and coverage completeness by Illumina short reads.


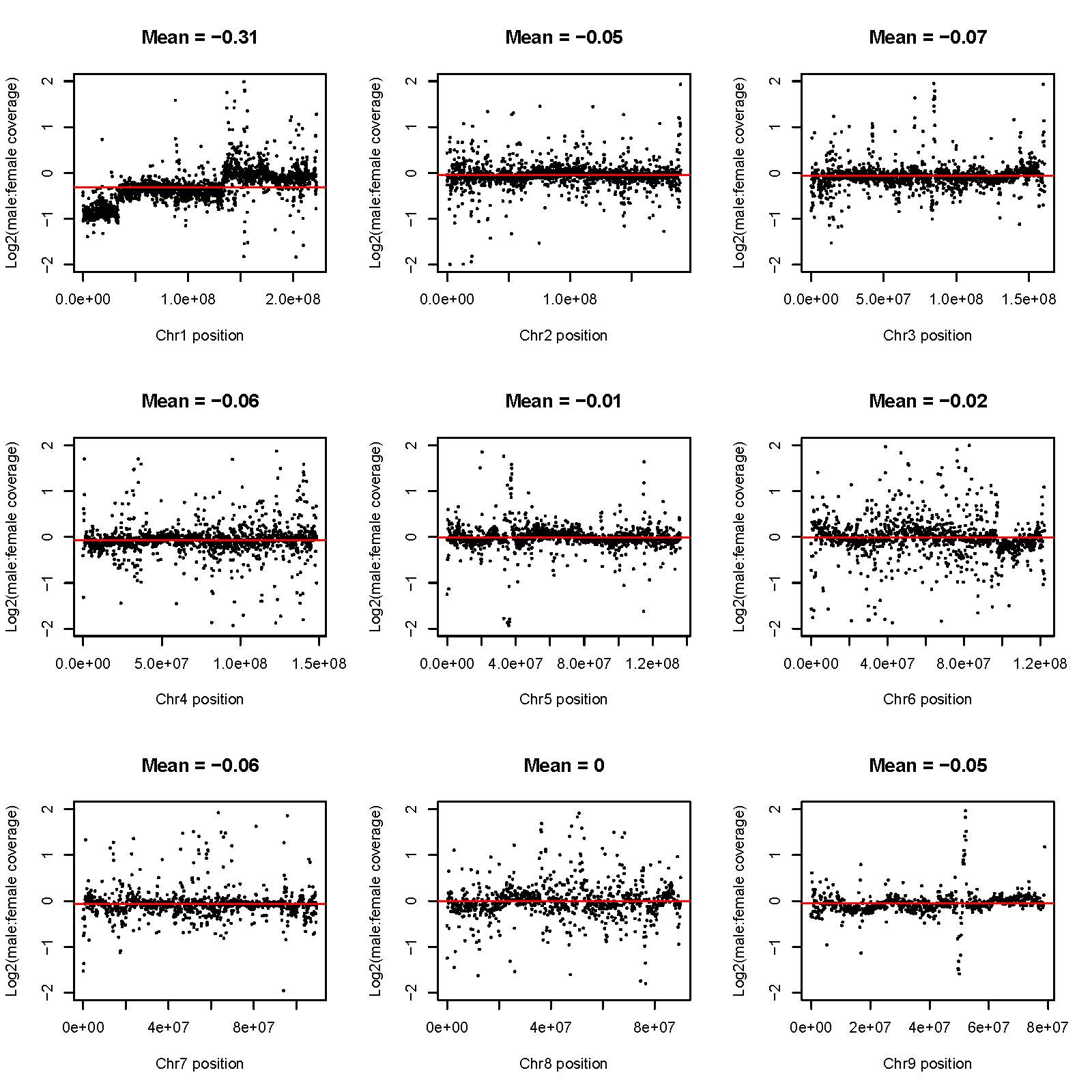


# Figure S4 Male:female coverage ratios plotted in 100 kb windows across each chromosome of the *O. nipae* genome.


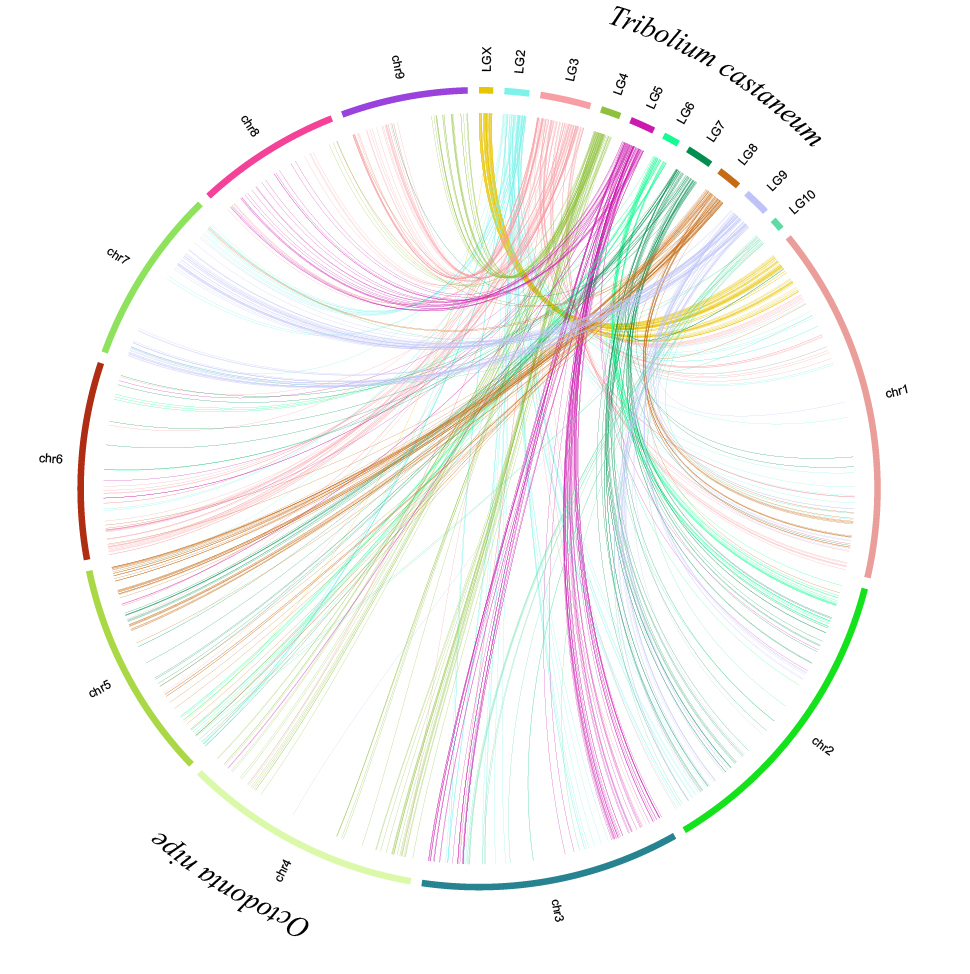


# Figure S5 Genom-wide synteny between *O. nipae* and *Tribolium castaneum* genomes.

# Table S1 Genome size and heterozygosity assessment via K-mer analysis

| **K-mer** | **K-mer number** | **K-mer depth** | **Genome size (Mbp)** | **Revised genome size (Mbp)** | **Heterozygous ratio (%)** | **Repeat (%)** |
| --- | --- | --- | --- | --- | --- | --- |
| 21 | 69,231,882,499 | 56 | 1,236.28 | 1,203.42 | 0.62 | 45.71 |

# Table S2 BUSCO evaluation result for the genome assembly of *O. nipae*

| **Species** | **Complete (%)** | **Fragmented (%)** | **Missed genes (%)** |
| --- | --- | --- | --- |
| *Octodonta nipae* | C:98.9%[S:85.7%,D:13.2%] | 0.20% | 0.90% |
| *Dendroctonus valens* | C:95.2%[S:86.6%,D:8.6%] | 1.00% | 3.80% |
| *Dendroctonus ponderosae* | C:95.3%[S:89.7%,D:5.6%] | 3.00% | 1.70% |
| *Tribolium castaneum* | C:99.3%[S:98.8%,D:0.5%] | 0.40% | 0.30% |
| *Aethina tumida* | C:99.5%[S:97.4%,D:2.1%] | 0.00% | 0.50% |

# Table S3 Genome data for comparative genomics of 21 coleoptera species

| **Organism Name** | **Order** | **Family** | **Number of Genes** | **Accession** |
| --- | --- | --- | --- | --- |
| *Anoplophora glabripennis* | Coleoptera | Cerambycidae | 14,828 | GCF_000390285.2 |
| *Diabrotica virgifera* | Coleoptera | Chrysomelidae | 19,526 | GCF_917563875.1 |
| *Diorhabda carinulata* | Coleoptera | Chrysomelidae | 11,977 | GCF_026250575.1 |
| *Diorhabda sublineata* | Coleoptera | Chrysomelidae | 11,704 | GCF_026230105.1 |
| *Phaedon cochleariae* | Coleoptera | Chrysomelidae | 13,141 | GCA_918026855.4 |
| *Phyllotreta striolata* | Coleoptera | Chrysomelidae | 11,862 | GCA_918026865.1 |
| *Psylliodes chrysocephala* | Coleoptera | Chrysomelidae | 15,767 | GCA_927349885.1 |
| *Coccinella septempunctata* | Coleoptera | Coccinellidae | 14,769 | GCF_907165205.1 |
| *Harmonia axyridis* | Coleoptera | Coccinellidae | 13,899 | GCF_914767665.1 |
| *Anthonomus grandis* | Coleoptera | Curculionidae | 13,112 | GCF_022605725.1 |
| *Ceutorhynchus assimilis* | Coleoptera | Curculionidae | 14,642 | GCA_917834065.1 |
| *Dendroctonus ponderosae* | Coleoptera | Curculionidae | 12,777 | GCF_020466585.1 |
| *Sitophilus oryzae* | Coleoptera | Curculionidae | 15,057 | GCF_002938485.1 |
| *Photinus pyralis* | Coleoptera | Lampyridae | 20,647 | GCF_008802855.1 |
| *Aethina tumida* | Coleoptera | Nitidulidae | 13,131 | GCF_024364675.1 |
| *Brassicogethes aeneus* | Coleoptera | Nitidulidae | 13,381 | GCA_921294245.1 |
| *Onthophagus taurus* | Coleoptera | Scarabaeidae | 14,537 | GCF_000648695.1 |
| *Nicrophorus vespilloides* | Coleoptera | Silphidae | 12,642 | GCF_001412225.1 |
| *Tenebrio molitor* | Coleoptera | Tenebrionidae | 21,414 | GCA_907166875.3 |
| *Tribolium castaneum* | Coleoptera | Tenebrionidae | 12,875 | GCF_000002335.3 |
| *Tribolium madens* | Coleoptera | Tenebrionidae | 12,167 | GCF_015345945.1 |
